# Prevalence and predictors of hypertension among patients undergoing preanesthesia evaluation at Bahir Dar University Tibebe Ghion Specialized Hospital in 2025: a cross-sectional study

**DOI:** 10.1186/s13741-026-00681-6

**Published:** 2026-04-18

**Authors:** Esubalew Muluneh Aligaz, Samuel Belay Ayalew, Sitotaw Tesfa Zegeye, Eshetu Tesfaye Dejen, Meseret Hulualem Nega, Zekarias Markos, Addisu Mequanint, Temesgen Mamo, Sintayehu Samuel Lorato

**Affiliations:** 1 Department of Anesthesia, Bahir Dar University College of Medicine and Health Science, Bahir Dar, Ethiopia; 2 Department of Anesthesia, Wachemo University College of Medicine and Health Science, Hossana, Ethiopia

**Keywords:** Hypertension, Surgical patients, Associated factors, Perioperative care

## Abstract

**Background:**

Hypertension is a major modifiable risk factor for perioperative morbidity and mortality, particularly in low- and middle-income countries where the burden of chronic disease is rising. Understanding its determinants among surgical patients is essential for improving perioperative safety and optimizing clinical outcomes.

**Methods:**

A cross-sectional study was conducted among 318 adult patients who were planned to undergo surgery. Socio-demographic, behavioral, clinical, and perioperative characteristics were collected using a questionnaire and chart review. Hypertension status was determined using standard blood pressure measurements. Logistic regression analyses were performed to identify factors associated with hypertension, and adjusted odds ratios (AOR) with 95% confidence intervals (CI) were reported. Statistical significance was set at *p* < 0.05.

**Results:**

The prevalence of hypertension among patients evaluated in the preoperative anesthesia clinic was 26.7% (95% CI: 21.9%–32.0%). Older age, behavioral factors, and clinical status were significantly associated with hypertension. Patients aged > 60 years had higher odds of hypertension compared to younger adults. Modifiable lifestyle factors including high salt intake (AOR = 2.3; 95% CI: 1.98–5.54), regular physical exercise (AOR = 0.49; 95% CI: 0.34–0.96), smoking (AOR = 2.73; 95% CI: 1.87–4.49), and current alcohol use (AOR = 1.6; 95% CI: 1.42–5.3) were significantly associated with hypertension. Comorbidity was associated with increased odds of hypertension (AOR = 2.58; 95% CI: 1.34–4.96).

**Conclusion:**

The overall prevalence of hypertension among surgical patients was found to be high. Its occurrence is strongly associated with modifiable lifestyle behaviors, existing comorbidities, and higher ASA status.

## Introduction

Hypertension is a major global public-health problem and a leading risk factor for cardiovascular disease, stroke, and premature death. The World Health Organization estimates that well over a billion adults worldwide have elevated blood pressure, and the absolute number continues to rise because of population ageing and epidemiologic transitions in low- and middle-income countries. Uncontrolled hypertension is associated with increased perioperative morbidity and mortality, principally through its effects on myocardial ischemia, heart failure, cerebrovascular events, and unstable hemodynamic responses to anesthetic drugs and surgical stress (Organization 2023).

The preanesthesia evaluation (preoperative assessment clinic or pre-anesthesia clinic) is the primary point at which previously undiagnosed or suboptimally controlled hypertension is detected and managed before elective and urgent procedures. Identification of elevated blood pressure during this evaluation has three simultaneous purposes: (Organization 2023) to stratify perioperative cardiovascular risk (Unger et al. 2020), to guide immediate perioperative anesthetic planning (choice of agents, monitoring, and intraoperative blood-pressure targets), and (Mills et al. 2011) to initiate or optimize long-term antihypertensive care when appropriate. As such, the prevalence and characteristics of hypertension in preanesthesia populations influence perioperative workflows, resource allocation (e.g., need for cardiology consultation, ambulatory blood-pressure monitoring), and institutional policies on cancellation or postponement of surgery for poorly controlled blood pressure (Unger et al. 2020).

Reported prevalence of hypertension in patients attending preanesthesia clinics varies substantially between settings, driven by differences in (a) the underlying population (age, comorbidity, urban vs. rural), (b) the threshold used to define hypertension (for example, the 2017 ACC/AHA threshold of ≥ 130/80 mmHg vs. older thresholds of ≥ 140/90 mmHg), (c) whether diagnosis is based on a single in-clinic measurement or on out-of-clinic/ambulatory blood-pressure monitoring (which reduces misclassification due to white-coat hypertension), and (d) sampling frame (general surgical population vs. specialty clinics such as vascular or cardiac surgery where hypertension prevalence is higher). Consequently, single-center studies have reported prevalence rates ranging from single digits in young low-risk cohorts to more than one-third in older or medically complex cohorts; systematic aggregation is therefore necessary to derive reliable local or regional estimates (Mills et al. 2011).

From a perioperative safety perspective, it is important to distinguish between chronic, well-controlled hypertension, transiently elevated clinic readings (white-coat hypertension), and true uncontrolled hypertension. Each carries different implications: chronic well-controlled hypertension generally permits proceeding with planned anesthesia while maintaining usual antihypertensive therapy (with selected exceptions, e.g., recent angiotensin-converting enzyme inhibitor continuation decisions), whereas newly detected or markedly elevated pressures may require further evaluation (cardiac risk stratification, assessment for end-organ damage) before elective surgery or targeted perioperative management if surgery cannot be delayed. Furthermore, perioperative blood-pressure variability and intraoperative hypertensive episodes are independently associated with adverse outcomes, reinforcing the need for careful preoperative recognition and optimization (Travieso-Gonzalez et al. 2019).

Despite the clinical importance, several knowledge gaps remain. First, many published prevalence estimates derive from single-center or high-income country cohorts; there is limited pooled evidence quantifying the burden of hypertension specifically at preanesthesia evaluations across different world regions and across surgical specialties. Second, heterogeneity in diagnostic criteria and measurement methods complicates comparison and meta-analysis. Third, the downstream consequences of detecting hypertension in the preanesthesia setting, such as rates of surgical delay, specialist referral, initiation of long-term antihypertensive therapy, or impact on perioperative outcomes, are incompletely characterized. Addressing these gaps is essential to inform pragmatic, evidence-based policies for preoperative blood-pressure screening, thresholds for action, and integration of perioperative detection into broader public-health hypertension control strategies.

### Aim of the present study

This study aims to determine the prevalence of hypertension among patients attending preanesthesia evaluation in Tibebe Ghion specialized hospitals, Ethiopia, to describe the characteristics (newly diagnosed vs. known; controlled vs. uncontrolled), and to examine how prevalence varies by age group, sex, and surgical specialty. Secondary objectives are to quantify the proportion of patients for whom surgery was postponed or who required additional perioperative evaluation because of elevated blood pressure, and to assess adherence to local or international preoperative blood-pressure management recommendations.

## Methods

### Study design and period

A hospital-based cross-sectional study was conducted from March 21, 2024 to May 21, 2025, to determine the prevalence and determinants of hypertension detected during preanesthesia evaluation.

### Study area

The study was conducted at Tibebe Ghion Specialized Hospital (TGSH), which is located in Bahir-Dar City, the capital of the Amhara Region, Northwest Ethiopia. The hospital was inaugurated on November 10, 2018, and operates under Bahir-Dar University as one of its major teaching and referral hospitals. TGSH is a tertiary-level institution providing a wide range of specialized medical and surgical services to the population of Bahir-Dar and its surrounding districts.

The hospital is equipped with modern diagnostic and treatment facilities and serves as a clinical training site for medical, anesthesia, nursing, and other health science students. The Department of Anesthesiology at TGSH runs a dedicated preanesthesia evaluation clinic, where patients scheduled for both elective and emergency surgeries undergo preoperative assessment and optimization. This setting made TGSH an appropriate site for studying the prevalence and determining factors of hypertension detected during preanesthesia evaluation.

### Population

#### Source population

All patients who attended the preanesthesia evaluation clinic of TGSH during the study period.

#### Study population

The study included all adult patients (≥ 18 years) who attended the preanesthesia clinic at Tibebe Ghion Specialized Hospital (TGSH) during the study period and provided informed consent. Eligible participants were those scheduled for elective or emergency surgery with complete evaluation data. Patients with severe psychiatric illness, incomplete records, or inability to communicate were excluded.

### Sample size and sampling technique

#### Sample Size determination

The sample size was calculated using the single population proportion formula, assuming a prevalence of hypertension of 25.1% based on a previous study in Bahir Dar (Anteneh et al. 2015), a 95% confidence level (Z = 1.96), and a 5% margin of error.

After adding a 10% non-response rate, the final sample size was 318 participants.

#### Sampling procedure

A systematic random sampling technique was used. Based on an estimated total of 600 patients attending the clinic during the study period, the sampling interval (K) was calculated as 600/318 ≈ 2. The first participant was randomly selected from the first two records, and subsequent participants were chosen at every second interval until the sample size was reached.

### Inclusion and exclusion criteria

#### Inclusion criteria

All adult patients aged ≥ 18years who attended the preanesthesia clinic at Tibebe Ghion Specialized Hospital (TGSH) for elective surgical procedures during the study period were included in the study.

#### Exclusion criteria

Patients who were unable to communicate effectively or had severe psychiatric illness were excluded from the study. Additionally, emergency surgeries requiring immediate intervention, where there was insufficient time for preoperative assessment and consent, were excluded.

### Variables of the study

#### Dependent variable

Prevalence of hypertension.

#### Independent variables

Socio-demographic: age, sex, marital status, residence (urban/rural), educational status, occupation, BMI, height, weight.

Behavioral/Lifestyle factors: smoking status, alcohol consumption, diet, and physical activity.

Clinical/Surgical factors: ASA physical status, type of surgery, comorbidities, family history of medical illness.

### Data collection methods

Data were collected using a structured and pretested checklist prepared in English to ensure consistency and completeness. The tool consisted of sections on socio-demographic characteristics, medical history, knowledge of hypertension, awareness of hypertension risk, lifestyle and behavioral factors, and clinical measurements relevant to hypertension. Patients’ knowledge of hypertension was assessed using a structured questionnaire containing several knowledge-related items addressing the definition, risk factors, prevention, and complications of hypertension. Each correct response was assigned one point, while incorrect or “don’t know” responses were scored as zero. The total knowledge score was calculated for each participant, and participants scoring equal to or above the mean value were categorized as having good knowledge, while those scoring below the mean were categorized as having poor knowledge. Eligible patients were consecutively screened and enrolled during the study period, and each participant was assessed once during their preanesthesia evaluation. Any missing or unclear information was clarified immediately with the patient or verified using medical records.

Data collection was carried out by two trained health workers who received instruction on the study objectives, the data collection tool, blood pressure measurement techniques, and ethical procedures. Their work was supervised by the principal investigator to ensure adherence to the study protocol.

Blood pressure (BP) measurements were obtained following standardized guidelines to ensure accuracy and reliability. During the procedure, participants were seated comfortably with their back supported, feet flat on the floor, and either arm placed at heart level. Before measurements were taken, each participant rested quietly for 5–10 min. BP was primarily measured using a digital BP device equipped with appropriate cuff sizes for each participant. Two readings were taken at least three minutes apart, and the mean systolic and diastolic BP values from these two measurements were used for analysis. Hypertension was defined as systolic BP ≥ 130 mmHg and/or diastolic BP ≥ 80 mmHg.

The questionnaire also captured behavioral and lifestyle characteristics, including dietary practices, physical activity, and knowledge and beliefs regarding hypertension. Clinical information from the preanesthesia evaluation was incorporated to determine the presence or absence of hypertension.

### Data quality control

Data quality was maintained through several rigorous measures implemented before and during data collection. To ensure the accuracy and reliability of the instrument, 5% of the questionnaires were pretested before the actual data collection period. Feedback from the pretest was used to refine the clarity, flow, and relevance of the items.

Data collectors and supervisors received comprehensive training on the study objectives, the data collection procedures, ethical considerations, and proper techniques for obtaining clinical measurements. The completeness and consistency of the collected data were reviewed daily, and any unclear or missing information was immediately clarified with participants or cross-checked with medical records.

To further enhance data quality, two professionals in the relevant field independently reviewed the completed questionnaires for completeness and logical accuracy. Continuous supervision throughout the data collection period ensured adherence to the study protocol and allowed early detection and correction of incomplete or erroneous entries.

For statistical analyses, the Hosmer–Lemeshow goodness-of-fit test was used to assess the adequacy of the logistic regression model. All statistical tests were two-sided, and a p-value of ≤ 0.05 was considered statistically significant. Odds ratios with their corresponding 95% confidence intervals were reported. All analyses were performed in accordance with the SAMPL (Statistical Analyses and Methods in the Published Literature) reporting guidelines.

### Data processing and analysis

The collected data were first checked for completeness, coded, and entered into Kobo Toolbox, then exported to SPSS version 26 for analysis. Descriptive statistics were used to summarize the data: categorical variables were presented as frequencies and percentages, while continuous variables were summarized using means and standard deviations.

Bivariable logistic regression analysis was initially performed to identify candidate variables associated with the outcome variable. Variables with a p-value < 0.25 in the bivariable analysis were considered for inclusion in the multivariable logistic regression model to control for potential confounding effects. Subsequently, multivariable logistic regression analysis was conducted to identify independent predictors of the outcome. The strength of the associations was measured using adjusted odds ratios (AORs) with 95% confidence intervals (CIs). A p-value < 0.05 was considered statistically significant.

Model fitness was assessed using the Hosmer–Lemeshow goodness-of-fit test, which indicated an adequate model fit. Multicollinearity among independent variables was evaluated using the Variance Inflation Factor (VIF), and no evidence of significant multicollinearity was observed (VIF < 2.5). The final model results were presented in tables with corresponding AORs, confidence intervals, and p-values.

### Operational definitions

The updated definition of hypertension, as per the 2025 American College of Cardiology (ACC) and American Heart Association (AHA) guidelines, categorizes blood pressure as follows (Jones et al. 2025).

Normal: Systolic BP < 120 mm Hg and Diastolic BP < 80 mm Hg.

Elevated: Systolic BP 120–129 mm Hg and Diastolic BP < 80 mm Hg.

Stage 1 Hypertension: Systolic BP 130–139 mm Hg or Diastolic BP 80–89 mm Hg.

Stage 2 Hypertension: Systolic BP ≥ 140 mm Hg or Diastolic BP ≥ 90 mm Hg.

Severe Hypertension: Systolic BP > 180 mm Hg and/or Diastolic BP > 120 mm Hg.

#### Good knowledge

Participants who correctly answered ≥ 50% of the knowledge-related questions regarding Hypertension were classified as having good knowledge (Organization 2013, Go et al. 2014).

#### Poor knowledge

Participants who correctly answered < 50% of the knowledge-related questions regarding Hypertension were classified as having poor knowledge (Organization 2013, Go et al. 2014).

## Results

### Socio-demographic characteristics

A total of 318 surgical patients were included in the study. Females accounted for the majority of participants (60.4%). The age distribution indicated that most participants were middle-aged and older adults, with the largest proportion aged 50–59 years, followed by those older than 60 **years**, while a relatively small proportion were aged 20–29 years.

Participants were almost equally distributed between urban and rural residences. Regarding marital status, the majority were married, whereas smaller proportions were single or divorced.

In terms of educational status, most participants had completed secondary or primary education, while a smaller proportion were uneducated or had attained tertiary education. With respect to occupation, merchants represented the largest occupational group, followed by daily laborers, farmers, and government employees.

Regarding preoperative physical status, most participants were classified as ASA physical status I, while smaller proportions were categorized as ASA II and ASA III (Table 1).

**Table 1:** Socio-demographic characteristics and clinical characteristics of surgical patients at Tibebe Ghion Specialized Hospital (TGSH), Bahir Dar, Ethiopia, 2025

Variables	Frequency	Percent
Sex		
Male	126	39.6
Female	192	60.4
Age groups		
20–29	26	8.2
30–39	66	20.8
40–49	69	21.7
50–59	85	26.7
> 60	72	22.6
Residence		
Urban	157	49.4
Rural	161	50.6
Marital status		
Single	80	25.2
Married	208	65.4
Divorced	30	9.4
Educational level		
Uneducated	63	19.8
Primary	122	38.4
Secondary	130	40.9
Tertiary	39	12.3
Occupation		
Government employed	43	13.5
Merchant	103	32.4
Daily laborer	89	28
Farmer	54	17
Others	29	9.1
ASA classification		
I	202	63.5
II	75	23.6
III	41	12.9
Monthly household income (ETB)		
< 5000	73	23
5000–10,000	150	47.2
> 10,000	95	29.9

### Prevalence of hypertension in surgical patients

The prevalence of hypertension among patients evaluated in the preoperative anesthesia clinic was 26.7% (95% CI: 21.9%–32.0%). In addition, 15% (95% CI: 10.1%–20.0%) demonstrated prehypertension that required further clinical attention. Overall, this indicates that approximately one in four surgical patients presented with blood pressure levels suggestive of hypertension during preoperative assessment.

20 patients (6.2%) had their surgeries postponed due to elevated blood pressure. This indicates that perioperative hypertension is a clinically significant issue within the surgical population, leading to delays in planned procedures. Postponement often occurs when blood pressure exceeds safe thresholds or is uncontrolled.

The mean systolic blood pressure among the study participants was 119.63 mmHg (SD ± 17.69), while the mean diastolic blood pressure was 78.73 mmHg (SD ± 8.95). No cases of isolated systolic hypertension or isolated diastolic hypertension were identified in the study population.

### Factors associated with hypertension in surgical patients

After controlling for potential confounders, several variables remained significantly associated with hypertension. Participants aged above 60 years had significantly higher odds of being hypertensive compared to those aged 20–29 years (*p* = 0.01). Individuals who reported using salt were more than twice as likely to have hypertension, with an adjusted odds ratio of 2.3 (95% CI: 1.98–5.54, *p* = 0.002). Regular physical exercise showed a protective effect, reducing the likelihood of hypertension by approximately 51% (AOR = 0.49, 95% CI: 0.34–0.96, *p* = 0.04). Patients with comorbid conditions also had significantly higher odds of hypertension (AOR = 2.58, 95% CI: 1.34–4.96, *p* = 0.04).

Behavioral factors, including smoking and current alcohol consumption, were also significant predictors; smokers had almost three times the odds of hypertension (AOR = 2.73, 95% CI: 1.87–4.49, *p* = 0.01), while alcohol drinkers had a 1.6-fold increased risk (AOR = 1.6, 95% CI: 1.42–5.3, *p* = 0.02).

Interestingly, Participants with poor blood-pressure knowledge had significantly higher odds of hypertension compared with those with adequate knowledge (AOR = 2.76; 95% CI: 1.50–3.20; *p* = 0.01), and those who were aware of hypertension risks also had higher odds of the condition (AOR = 3.10, 95% CI: 2.12–4.92, *p* = 0.04), likely reflecting increased exposure to health information following a diagnosis (Table 2).

**Table 2:** Hypertension and associated factors among surgical patients at Tibebe Ghion Specialized Hospital (TGSH), Bahir Dar, Ethiopia, 2025

Variable	COR (95%CI)	AOR(95%CI)	*P*- value
Sex			
Male	1		
Female	1.24(0.75–2.06)		0.98
Age groups			
20–29	1		
30–39	1.97(0.66–5.87)		0.78
40–49	1.64(0.79–3.38)		0.56
50–59	1.45(0.72–2.95)		0.57
>60	1.49(1.23–4.94)	2.12(1.05–4.27)	0.01*
Salt use			
Yes	2.52(1.42–4.45)	2.3(1.98–5.54)	0.002*
No	1		
Physical exercise			
Yes	0.39(0.22–0.70)	0.49(0.34–0.96)	0.04*
No	1	1	
Family history of HTN			
Yes	2.52(1.42–4.45)	2.94(2.1–4.99)	0.001*
No	1	1	
BMI			
Normal	1	1	
Overweight/obese	2.20(1.28–3.94)	7.0(5.45–10.1)	0.001*
Comorbidity			
Yes	4.27(2.55–7.30)	2.58(1.34–4.96)	0.04*
No	1		
Type of surgery			
General surgery	1		
Gynecological surgery	1.08(0.53–2.19)		0.67
ENT	0.97(0.50–1.88)		0.86
ASA class			
I	1		
II	8.76(4.6–16.4)	8.9(5.1–17.2)	0.01*
III	5.70(2.73–11.8)	7.2(2.23–19.1)	0.01*
Smoking			
Yes	2.0(1.42–4.45)	2.73(1.87–4.49)	0.01*
No	1		
Current Alcohol drinking			
Yes	1.52(1.12–4.45)	1.6(1.42–5.3)	0.02*
No	1		
Blood pressure knowledge			
Adequate	1	1	
Poor	1.72(1.42–4.45)	2.76(1.5–3.20)	0.01*
Risk hypertension awareness			
Yes	2.20(1.42–4.45)	3.10(2.12–4.92)	0.04*
No	1		

*p* < 0.05 (bolded) and * indicates statistically significant associations

*Abbreviations*: *AOR* Adjusted odds ratio, *CI* Confidence interval, *COR* Crude odds ratio

## Discussion

This study assessed the prevalence and determinants of hypertension among surgical patients and identified several significant socio-demographic, behavioral, and clinical predictors. The findings demonstrate that hypertension is strongly associated with older age, high salt intake, physical inactivity, comorbidities, higher ASA classification, smoking, alcohol consumption, and levels of blood pressure knowledge and risk awareness. These patterns are consistent with established pathophysiological mechanisms and with evidence from previous studies in Ethiopia and other low- and middle-income countries (Schutte et al. 2021, Owolabi et al. 2016, Tiruneh et al. 2020, Tadesse et al. 2025).

In this study, the prevalence of hypertension among patients evaluated in the preoperative anesthesia clinic was 26.7%, demonstrating that more than one-quarter of surgical candidates had elevated blood pressure requiring perioperative attention. This prevalence is consistent with reports from similar perioperative settings, where hypertension is recognized as the most common medical comorbidity encountered before surgery (Wijeysundera et al. 2014). The identification of hypertension at this stage is clinically important, as poorly controlled blood pressure increases the likelihood of intraoperative hemodynamic fluctuations, myocardial ischemia, and postoperative complications (Whelton and Williams 2018).

The study also found that 15% of patients had prehypertension, indicating an additional group at increased risk for future cardiovascular disease. This underscores the valuable role of preoperative clinics in detecting undiagnosed or subclinical hypertension, which aligns with previous findings that preoperative visits often serve as an entry point for cardiovascular screening and referral (Bijker and Agyemang 2016). Early detection allows for lifestyle counseling and timely follow-up, potentially reducing long-term cardiovascular risk.

In this study, 20 patients (6.2%) had their elective surgeries postponed due to elevated preoperative blood pressure. This finding underscores the significance of perioperative hypertension as a common barrier to timely surgical care and its role as a modifiable risk factor for adverse outcomes. Hypertension is one of the most frequently encountered medical conditions in preoperative clinics, and its presence has been associated with increased perioperative cardiovascular risk. Markedly elevated blood pressure is known to predispose patients to intraoperative hemodynamic instability, myocardial ischemia, arrhythmias, stroke, and postoperative complications such as heart failure and hypertensive crises (Howell et al. 2004). A large retrospective cohort study additionally demonstrated that elevated pre-induction systolic blood pressure is independently associated with myocardial injury and higher in-hospital mortality (Sabaté et al. 2011). These risks justify postponement in cases of severe or uncontrolled hypertension.

Despite the prevalence of hypertensive and prehypertensive cases, the mean systolic (110.63 mmHg) and diastolic (78.73 mmHg) pressures for the total population remained within the normal range. Furthermore, the absence of isolated systolic or diastolic hypertension suggests that most hypertensive cases involved combined elevation of both parameters, a pattern more commonly observed in adults undergoing elective surgical procedures (Ueshima et al. 2017).

Age was one of the strongest predictors of hypertension. Participants older than 60 years had significantly higher odds of being hypertensive compared with younger adults. This aligns with findings from studies in Ethiopia, Kenya, Nigeria, and other sub-Saharan African settings, where age-related vascular changes, increased arterial stiffness, endothelial dysfunction, and cumulative exposure to cardiovascular risk factors increase susceptibility to hypertension (Odili et al. 2020, Ogungbe et al. 2024, Olack et al. 2015, Kebede et al. 2020). The lack of significance among the intermediate age groups (30–59 years) after adjustment may indicate overlapping risk exposures or the confounding influence of comorbid conditions that are more common in the oldest age category (Legese and Tadiwos 2020).

Lifestyle behaviors showed a clear influence on blood pressure status. High salt intake significantly increased the odds of hypertension, which is consistent with WHO recommendations linking sodium consumption to elevated blood pressure (Kario et al. 2024). The strong effect observed in this study reinforces the importance of dietary modification, especially in perioperative patients who already face increased cardiovascular stress (Jones et al., Ji-Guang 2025). In contrast, engagement in regular physical exercise reduced the likelihood of hypertension by nearly half, consistent with evidence that physical activity improves endothelial function, reduces sympathetic overactivity, and lowers systemic vascular resistance (Olowoyo et al. 2024).

Comorbidity was another key determinant, with patients who had additional medical illnesses more than twice as likely to be hypertensive (Huda et al. 2025). Chronic conditions such as diabetes mellitus, chronic kidney disease, and cardiovascular disorders frequently coexist with hypertension, contributing to a cycle of worsening metabolic and hemodynamic dysfunction (Balgobin et al. 2024). The strong association between hypertension and ASA classification further supports this relationship. Patients categorized as ASA II and III had markedly higher odds of hypertension compared to those in ASA I, reflecting the cumulative effect of systemic diseases on cardiovascular status. These findings are in line with previous perioperative studies showing that hypertension often clusters with other chronic illnesses and is a major contributor to higher ASA scores (Koutsaki et al. 2019).

Behavioral risk factors, including smoking and alcohol consumption, also demonstrated significant associations. Smokers had almost three times the risk of hypertension, consistent with evidence linking tobacco use to increased arterial stiffness, sympathetic activation, and impaired vasodilation. Similarly, current alcohol consumption was associated with higher odds of hypertension, reflecting established physiological effects of alcohol on catecholamine release, renin–angiotensin system activation, and vascular remodeling (Pantell et al. 2019, Sisay et al. 2022).

Our study found participants with better knowledge of blood pressure and greater awareness of hypertension risk had lower odds of hypertension compared with those with poor knowledge (Aung et al. 2012). Individuals who understood hypertension, its risk factors, and prevention strategies were less likely to be hypertensive than those with poor knowledge (Agyei-Baffour et al. 2018). This suggests that health literacy plays a protective role by promoting healthy behaviors such as reducing salt intake, maintaining physical activity, and seeking regular screening. Risk-aware individuals are also more likely to adopt preventive practices and recognize early warning signs, contributing to reduced hypertension incidence. These findings highlight the importance of strengthening community-based health education to improve population-level awareness and prevent hypertension before it develops (Eghbali et al. 2018).

### Strength of the study

This study represents the first evidence from Ethiopia to describe the prevalence and predictors of hypertension among patients undergoing preoperative evaluation, providing important context-specific insights.

### Limitation of the study

This study has several limitations. The cross-sectional design limits the ability to establish causal relationships between the studied variables. In addition, the single-center nature of the study may reduce the generalizability of the findings to other settings. Blood pressure measurements obtained during the preanesthesia evaluation may have been influenced by transient factors such as patient anxiety (white-coat effect). Furthermore, the use of self-reported data may introduce recall bias, and residual confounding cannot be completely excluded. Finally, the exclusion of ASA IV patients, who often represent the highest-risk group for perioperative complications, may introduce selection bias and limit the external validity and generalizability of the findings to real-world high-risk surgical populations.

## Conclusion and recommendations

This study found a high prevalence of hypertension among patients undergoing preanesthesia evaluation, with more than one-quarter presenting with elevated blood pressure and an additional proportion demonstrating prehypertension. Hypertension was strongly associated with older age, high salt intake, physical inactivity, comorbid medical illness, higher ASA classification, smoking, alcohol consumption, overweight/obesity, and poor knowledge and awareness of hypertension. These findings highlight the combined influence of modifiable behavioral factors and chronic disease burden on hypertension among surgical patients.

Notably, 6.2% of patients had their surgeries postponed because of uncontrolled blood pressure, emphasizing the perioperative risk posed by hypertension and the need for early detection and optimization before surgery.

### Recommendations

In clinical practice, it is essential to strengthen preanesthesia hypertension screening by using repeated blood pressure measurements and ensuring earlier preoperative evaluation. Blood pressure should be optimized before elective surgery, particularly in patients with Stage 2 hypertension or evidence of end-organ involvement. Lifestyle counseling should be integrated into routine preoperative assessment, emphasizing salt reduction, regular physical activity, smoking cessation, moderation of alcohol intake, and weight management. Institutions should establish clear guidelines that define when to proceed with or postpone surgery.

From a hospital administration and policy perspective, the development of dedicated preoperative optimization pathways, including referral to medical or cardiology services for uncontrolled hypertension, is recommended. Training programs for anesthetists and nurses should be implemented to improve the accuracy of blood pressure measurement and enhance understanding of updated ACC/AHA guidelines. Hospitals should also promote hypertension education materials within surgical clinics to improve patient knowledge and encourage self-management.

At the public health level, strengthening community-based hypertension screening and awareness programs will help improve early detection and prevention. Routine blood pressure assessment in primary care settings should be encouraged to reduce the number of patients first diagnosed at the time of surgery.

For future research, multicenter prospective studies are needed to evaluate perioperative outcomes among hypertensive surgical patients in Ethiopia. Further research should also assess the effectiveness of preoperative optimization interventions in reducing surgical delays, perioperative complications, and long-term cardiovascular outcomes.

## Data Availability

The datasets used and analyzed during the current study are available from the corresponding authors upon reasonable request.

## Abbreviations

ACC/AHA: American College of Cardiology/American Heart Association
ASA: American Society of Anesthesiology
ENT: Ear Nose Throat
TGSH: Tibebe Ghion specialized hospital
WHO: World Health Organization
